# Temporal trend of tuberculosis incidence and its spatial distribution in Macapá – Amapá

**DOI:** 10.11606/s1518-8787.2021055003431

**Published:** 2021-11-18

**Authors:** Clóvis Luciano Giacomet, Marcio Souza Santos, Thaís Zamboni Berra, Yan Mathias Alves, Luana Seles Alves, Fernanda Bruzadelli Paulino da Costa, Antonio Carlos Vieira Ramos, Juliane de Almeida Crispim, Aline Aparecida Monroe, Ione Carvalho Pinto, Regina Célia Fiorati, Marcos Augusto Moraes Arcoverde, Dulce Gomes, Giselle Lima de Freitas, Mellina Yamamura, Ricardo Alexandre Arcêncio

**Affiliations:** I Universidade de São Paulo Escola de Enfermagem de Ribeirão Preto Ribeirão Preto SP Brasil Universidade de São Paulo. Escola de Enfermagem de Ribeirão Preto. Ribeirão Preto, SP, Brasil; II Universidade do Oeste do Paraná Escola de Enfermagem Foz do Iguaçu PR Brasil Universidade do Oeste do Paraná, Escola de Enfermagem. Foz do Iguaçu, PR, Brasil; III Universidade de Évora Departamento de Matemática Évora Portugal Universidade de Évora. Departamento de Matemática. Évora, Portugal; IV Universidades Federal de Minas Gerais Faculdades de Enfermagem Belo Horizonte MG Brasil Universidades Federal de Minas Gerais. Faculdades de Enfermagem. Belo Horizonte, MG, Brasil; V Universidades Federal de São Carlos Faculdades de Enfermagem São Carlos SP Brasil Universidades Federal de São Carlos. Faculdades de Enfermagem. São Carlos, SP, Brasil

**Keywords:** Tuberculosis, epidemiology, Space-Time Clustering, Spatial Analysis, Ecological Studies

## Abstract

**OBJECTIVE:**

To evaluate the temporal trend of tuberculosis incidence after the implementation of the rapid molecular test (RMT-TB), to identify whether tuberculosis presents seasonal variation and to classify the territory according to case density and risk areas in Macapá, Amapá.

**METHODS:**

Ecological study of tuberculosis cases registered in the *Sistema de Informação de Agravos de Notificação* (SINAN – Information System for Notifiable Diseases) between 2001 and 2017. We used the Prais-Winsten test to classify the temporal trend of incidence and the interrupted time series to identify changes in the temporal trend before and after the implementation of the rapid molecular test, and to verify seasonality in the municipality. The Kernel estimator was used to classify case density and scan statistics to identify areas of tuberculosis risk.

**RESULTS:**

A total of 1,730 cases were identified, with a decreasing temporal trend of tuberculosis incidence (−0.27% per month, 95%CI −0.13 to −0.41). The time series showed no change in level after the implementation of the GeneXpert®MTB/RIF molecular test; however, the incidence increased in the post-test period (+2.09% per month, 95%CI 0.92 to 3.27). Regarding the seasonal variation, it showed growth (+13.7%/month, 95%CI 4.71 to 23.87) from December to June, the rainy season – called amazon winter season –, and decrease (−9.21% per month, CI95% −1.37 to −16.63) in the other periods. We classified areas with high density of cases in the Central and Northern districts using Kernel and identified three protection clusters, SC1 (RR = 0.07), SC2 (RR = 0.23) and SC3 (RR = 0.36), and a high-risk cluster, SC4 (RR = 1.47), with the scan statistics.

**CONCLUSION:**

The temporal trend of tuberculosis incidence was decreasing in the time series; however, detection increased after the introduction of RMT-TB, and tuberculosis showed seasonal behavior. The case distribution was heterogeneous, with a tendency to concentrate in vulnerable and risk territories, evidencing a pattern of disease inequality in the territory.

## INTRODUCTION

According to the World Health Organization (WHO), tuberculosis is one of the top 10 causes of death in the world and the leading cause of death by a single infectious agent in people living with the human immunodeficiency virus (HIV), thus, remaining a worldwide public health problem. In 2019, about 10 million people became ill with tuberculosis worldwide and about 1.4 million people died as a result of the disease^1^.

In Brazil, in 2019, 73,864 new cases of tuberculosis were diagnosed (incidence of 35.0 cases/100,000 inhabitants) and note that the Northern region of the country is the most affected by the disease^2^. In the state of Amapá, in 2019, 296 new cases of the disease were diagnosed in 14 of its 16 municipalities, Macapá being the city with the highest number of cases, with a recorded increase of 23.9% compared to 2018^3^.

For its eradication, wider-ranging actions are necessary, such as the active search for respiratory symptoms aiming at the early diagnosis of cases and the rapid start of treatment, to prevent the tuberculosis-infected person from remaining in the bacilliferous phase and transmitting the disease to other people and communities^4^.

Regarding diagnosis, the rapid molecular test for tuberculosis (RMT-TB), performed by using the GeneXpert^®^ MTB/IfR system, consists of an automated examination, which reduces the time of diagnosis result to a maximum of 2 hours and saves on costs, including those from hospitalizations resulting from the disease evolution and the late diagnosis. Hospitalization costs due to tuberculosis increase by up to one hundred times in relation to those of outpatient treatment^5^.

With faster and highly sensitive and specific – respectively 98% and 88%^5^ – results, treatment can be started early, without the need to wait for confirmation by culture (which can take up to 60 days).

The WHO validated the RMT-TB results in 2010 after which the diagnostic technology was recommended and adopted in several health systems worldwide, including in Brazil, where the National Commission for the Incorporation of Technologies of the Unified Health System (CONITEC-SUS) and the National Health Surveillance Agency (ANVISA) approved the use of the test in 2013. The Ministry of Health acquired 160 Cepheid laboratory equipment and strategically distributed them throughout the country^5^.

We hypothesized that the RMT-TB, due to its high sensitivity, may have modified the number of diagnosed cases and contributed to the elucidation of cases, specifically paucibacillary cases, previously undetected by bacilloscopy. In other words, we investigated whether RMT-TB may have changed the epidemiological panorama of tuberculosis.

In addition to the increase in cases, another hypothesis of this study is the seasonal variation of the disease, a phenomenon that has been observed in studies conducted in several countries^6^, but little explored in Brazil. Another study^7^ showed that respiratory diseases increased in the Amazon in the winter periods, when rainfall in the region increases, therefore, the hypothesis arises that tuberculosis rates may also increase.

Considering the above, our study sought to evaluate the temporal trend of tuberculosis incidence after the implementation of RMT-TB, identify whether it presents seasonal variation and classify the territory according to case density and risk areas for the disease in a municipality of eastern Amazonia.

## METHODS

### Study Design

Ecological study^8^ conducted in Macapá, capital of the Brazillian state of Amapá, which is part of the Eastern Amazon and borders French Guiana.

### Place of Study

The city of Macapá has an area of approximately 6,563.849 km², a population density of 62.14hab/km² and an estimated population of 512,902 people in 2020. It is administratively divided into four districts: Northern, Western, Central and Southern^9^.

The diagnosis of tuberculosis cases in Macapá follows the protocol of the Ministry of Health^5^. Thus, all basic health units (UBS) receive suspected cases that are later referred to the state reference unit, called the Reference Center for Tropical Diseases (CRDT). It is a state-wide centralized service, which monitors cases and dispenses medicines.

The state also has the Central Laboratory (LACEN), which performs laboratory tests, including those for tuberculosis diagnosis. The only GeneXpert^®^ MTB/RIF equipment, a reference for the diagnosis of tuberculosis, in Amapá is in the LACEN. We emphasize that the RMT-TB was implemented in September 2014, which was considered the cut-off year in the time series^5^.

### Population

The study's population consisted of tuberculosis cases reported in the *Sistema de Informação de Agravos de Notificação* (SINAN – Information System for Notifiable Diseases) between 2001 and 2017.

### Analysis Plan

Initially, to characterize the profile of tuberculosis cases reported in the municipality under study, the absolute and relative frequencies of the variables presented in the notification form were calculated using the IBM SPSS Statistics software version 25.

To classify the temporal trend of tuberculosis incidence, the rate was calculated monthly, considering the absolute number of cases in the numerator and the population in the denominator, with multiplication factor per 1000 inhabitants. The incidence rates were converted into logarithms (log10) to stabilize variance over time^10^. The graphs of case distribution and of estimated rates throughout the time series were made using the RStudio software.

The Prais-Winsten self-regression method was used in the STATA software to classify the tuberculosis incidence temporal trend into increasing, decreasing or stationary and, if increasing or decreasing, the monthly percent change (MPC) and its respective 95% confidence interval (95%CI) was calculated^10^.

The interrupted time series (ITS) was used to verify whether the temporal trend of tuberculosis incidence changed after the implementation of RMT-TB and whether the disease presents seasonal variation. It is defined as the most effective resource to evaluate the impact of an intervention, allowing to verify whether there is immediate influence (change of level) and/or progressive influence (trend change) on the values of the series^11^.

For the application of the ITS, the previously calculated logarithmic monthly incidence rates (log10) were considered. The change in level was called “intervention” and the progressive change, “post-intervention”^11^. Trigonometric functions sine and cosine were also incorporated into the analyses to verify whether tuberculosis presents seasonal variation^10^. STATA software version 14 was used for ITS.

In the first stage, the inclusion criteria was all reported cases of tuberculosis from residents in the urban area of Macapá. For cases notified more than once in the system, the most up to date notification was considered. Note that, in the analyzed period, all cases listed in the study were from new notifications in SINAN, therefore, the incidence was estimated.

The Kernel density estimator was used to classify the density of cases. Initially, performing the geocoding of the cases by the geographic coordinates (latitude and longitude) of the residential addresses of the reported cases – obtained using the Google Earth Pro tool – was necessary.

The georeferencing of the cases included in the study was performed by the ArcGis 10.5 software, considering the urban census tracts of the municipality of Macapá as an analysis unit and the residing population by census tract, as a configuration for analysis standardization.

Note that, in the spatial analysis phase, cases of homeless people, individuals deprived of liberty, indigenous people or people whose notification address was from municipal agencies (hospitals or health units) were excluded due to the impossibility of obtaining the geographic coordinates of residential addresses.

The Kernel intensity estimator was used from the ArcGis 10.5 software. It is an exploratory interpolation method that defines circular areas of influence around occurrence points of a phenomenon, generating a surface density to identify vulnerable areas^12^. Thus, considering a radius of 1,000 meters^13,14^, thematic maps of the tuberculosis case density distribution were also generated in the ArcGIS 10.5 software.

Finally, to identify the risk areas for tuberculosis in Macapá, the scan statistic^15^ was used, in which a circle of variable radius around the centroide of each unit of analysis, which were the census tracts in this study, is used to search for spatial clusters. In this technique, the number of observed and expected cases within each circle is calculated beforehand, until all centroides are tested. When the observed value in the area inside the circle is greater or smaller than expected, the circle is called a cluster^16^.

In these analyses, the following characteristics were adopted: Poisson discrete model; circular-shaped agglomerates; no geographical overlap of the agglomerates; 999 replications in Monte Carlo simulation; and exposed population size stipulated by the Gini coefficient, in which the number of cases is compared to the data of the base population and the expected number of cases in each census tract is proportional to the size of the population at risk^16,17^. The relative risk (RR) and confidence interval (95%CI) of each cluster were also calculated. Note that clusters with p < 0.05 were considered statistically significant.

The study was approved by the Research Ethics Committee of the Escola de Enfermagem de Ribeirão Preto (CAAE No. 23043019.2.0000.5393).

## RESULTS

Between 2001 and 2017, 1,730 cases of tuberculosis were reported in Macapá, with a minimum age of one year and a maximum of 89 years.

Most cases were female (59.3%), aged between 31 and 59 years (47.6%), mixed race (67.9%) and incomplete elementary school education (46.6%). The pulmonary form is predominant (85.1%).

Table 1 shows the high frequency of unanswered and/or ignored information regarding schooling (16.4%), TB-HIV co-infection (50%), TB-Diabetes (41.8%), alcoholism (39.7%) and mental illness (41.7%), despite the relevance of this information for the clinical management of cases and conducts.

**Table 1:** Sociodemographic and epidemiological clinical profile of tuberculosis (TB) cases reported in Macapá, state of Amapá, Brazil (2001 to 2017).

Variables		n (1,730) (%)
Age (years)		
	0 to 14 years	74 (4.3)
	15 to 3 years	640 (37.0)
	31 to 59 years	823 (47.6)
	≥ 60 years	175 (10.1)
	Ignored/unanswered	18 (1.0)
Gender		
	Male	704 (40.7)
	Female	1,026 (59.3)
Race		
	White	325 (18.3)
	Black	124 (7.7)
	Asian	28 (1.6)
	Mixed race	1,175 (67.9)
	Indigenous	14 (0.8)
	Ignored/unanswered	64 (3.7)
Schooling level		
	Illiterate	128 (7.4)
	Complete elementary school	112 (6.5)
	Incomplete elementary school	803 (46.6)
	Complete secondary education	224 (12.9)
	Incomplete higher education	52 (3.0)
	Complete higher education	127 (7.3)
	Ignored/unanswered	284 (16.4)
Forms		
	Extrapulmonary	236 (13.6)
	Pulmonary	1,472 (85.1)
	Pulmonary + extrapulmonary	18 (1.0)
	Ignored/unanswered	4 (0.3)
Outcome		
	Cure	1,303 (75.3)
	Abandonment	217 (12.5)
	Death from tuberculosis	17 (1.0)
	Death from other cause	47 (2.7)
	Transfer/change of country	108 (6.3)
	Diagnosis change	30 (1.7)
	Multi-resistant TB	04 (0.2)
	Change of regimen by drug intolerance	03 (0.2)
	Diagnosis change	01 (0.1)
TB-HIV co-infection		
	No	787 (45.5)
	Yes	77 (4.5)
	Ignored/unanswered	866 (50.0)
TB-diabetes co-infection		
	No	912 (52.8)
	Yes	94 (5.4)
	Ignored/unanswered	724 (41.8)
Alcoholism		
	No	896 (51.7)
	Yes	148 (8.6)
	Ignored/unanswered	686 (39.7)
Mental disorder		
	No	1,001 (57.8)
	Yes	12 (0.7)
	Ignored/unanswered	717 (41.5)

**Figure 1:**
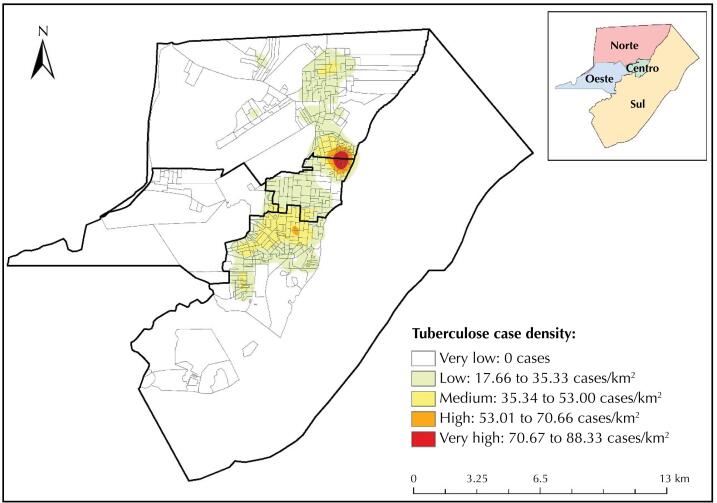
Time series of the number of cases and tuberculosis incidence in Macapá, Amapá, Brazil (2001 to 2017).

Figure 1 shows the monthly time series of the number of cases and incidence of tuberculosis from 2001 to 2017, in which positive and negative peaks occur throughout the study period. We concluded that the series begins with higher numbers, suffers a decrease and ends in an increase.

Table 2 shows the results of the Prais-Winsten and ITS techniques. The temporal trend of tuberculosis incidence in Macapá was classified as decreasing at a −0.27%/month rate (95%CI −0.41 to −0.13).

**Table 2:** Temporal trend, impact of diagnosis by rapid molecular testing and seasonal variation in the incidence of tuberculosis cases, Macapá, Amapá, Brazil (2001 to 2017).

Prais-Winsten			
	Coefficient (95%CI)	Trend	MPC (95%CI)
Tuberculosis in Macapá	-0.001 (−0.006 to −0.000)	Decreasing	-0.27 (−0.41 to −0.13)
**Interrupted time series (ITS)**			
	Coefficient (95%CI)	Trend	MPC (95%CI)
Intervention	1.014 (3.001 to −0.971)	Unchanged	N/A
Post-intervention	0.009 (0.014 to 0.004)	Increasing	2.09 (3.27 to 0.92)
Sine	0.056 (0.093 to 0.020)	Increasing	13.76 (23.87 to 4.71)
Cosine	-0.042 (−0.079 to −0.006)	Decreasing	-9.21 (−16.63 to −1.37)

MPC: *monthly percent change*;

N/A: not applicable

We saw no change of level (intervention) in the time series after the implementation of RMT-TB. However, we classified the period after the implantation of RMT-TB as increasing, with a 2.09%/month rate (95%CI 3.27 to 0.92), indicating that the incidence of tuberculosis increased progressively after the implementation of the test.

Regarding seasonality, the disease in Macapá showed a growth of 13.7%/month (95%CI 23.87 to 4.71) in the months between December and June (sine), a period that coincides with the rains of the so-called Amazonian winter. We saw a decrease of 9.21%/month (95%CI −1.37 to −16.63) in the remaining months (cosine), which are warmer and dryer seasons.

Of the 1,730 tuberculosis cases identified, 59 (3.41%) were excluded for lack of address. Of the remaining 1,671 cases, 1,475 (88.2%) had their geographic coordinates identified, thus georeferenced.

**Figure 2:**
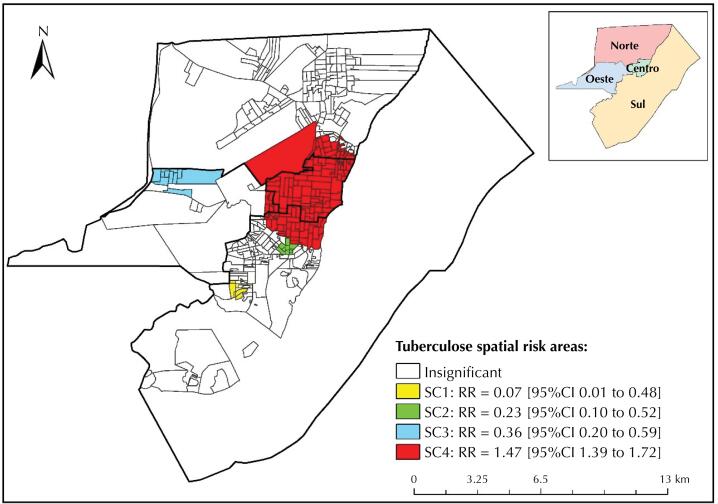
Geographic location of tuberculosis cases reported in Macapá-AP, Brazil (2001 to 2017).

With the Kernel estimator, we observed areas classified as very high density of cases in the Central (neighborhoods of Laguinho and Perpétuo Socorro) and Northern (neighborhoods of Pacoval and Cidade Nova) districts, ranging from 70.67 to 88.33 cases/km². Figure 2 shows that areas classified as high density of cases are in the Southern (neighborhood of Buritizal), Central (neighborhoods of Laguinho and Perpétuo Socorro) and Northern (neighborhoods of Pacoval and Cidade Nova) districts, ranging from 53.01 to 70.66 cases/km^2^.

**Figure 3:**
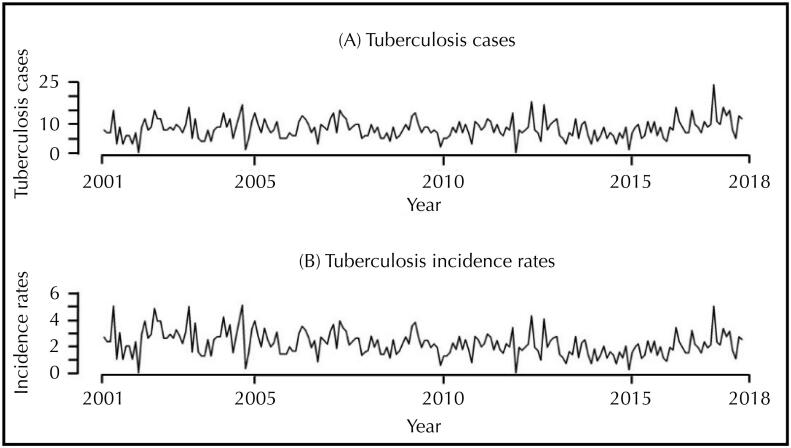
Areas of spatial risk for tuberculosis in Macapá, Amapá, Brazil (2001 to 2017).

Using the scan statistics, we identified four spatial clusters (SC) at risk for tuberculosis in Macapá (Figure 3), considering the parameter of 40% of the exposed population, according to the Gini index.

The SC1, considered a protection cluster (RR = 0.07; 95%CI 0.01 to 0.48), consisted of six census tracts (p = 0.03) of the Southern district, with a population of 3,799 inhabitants, one observed case and 14 expected cases.

The SC2, another protection cluster (RR = 0.23; 95%CI 0.10 to 0.52), consisted of 10 census tracts (p = 0.03) of the Southern district, with a population of 6,578 inhabitants, six observed cases and 25 expected cases.

The SC3, also considered protective (RR = 0.36; 95%CI 0.20 to 0.59), consisted of 11 census tracts of the Western district (p = 0.04), with 10,172 inhabitants, 14 observed cases and 38 expected cases.

Finally, SC4 (p < 0.01), considered a high-risk cluster for tuberculosis (RR = 1.47; 95%CI 1.39 to 1.72), consisted of 146 census tracts of the Northern, Southern and Central districts, with a population of 122,356 inhabitants, 620 observed cases and 487 expected cases.

## DISCUSSION

We managed to trace the sociodemographic and clinical-epidemiological profile of tuberculosis cases in Macapá, Amapá. By the calculation of the monthly incidence in a 16-year time series, we could classify the temporal trend and identify changes in the series after RMT-TB implementation, besides showing that tuberculosis has seasonal variation. Finally, we could classify areas according to the case density and identify risk and protection areas in Macapá, Amapá.

Most people affected by tuberculosis in Macapá were female, which differs from the findings of the literature that shows cases mostly in males – explained by the fact that men tend to minimize their self-care and be more exposed to risk factors for the disease^18^.

The predominance of women in this case may be related to the fact that women are heads of families, undervalued in the labor market and often work triple hours, which are stress factors that can be considered a risk for illness^18^. The gender inequality, inherited from local cultural issues of the riverside peoples in which women are seen as adjunct and the man as a provider, persists to this day and is another stress factor.

Most cases were detected in adults in an economically active age group, a phase of life in which individuals tend to face greater agglomerations, due to work and daily activities, increasing the chances of illness^19^.

Mixed race people were more prone to the disease in the study, which can be justified by being the predominant race/color in the municipality. Almost 52% of the Macapá population declares itself as mixed race and, in this study, the self-declared as mixed race are 67.9%, which would justify the high rates of tuberculosis in this population.

Most reported cases have incomplete elementary school, which is an indirect indicator of social conditions caused by the population's social context. This data represents an obstacle to TB control, since these individuals may have difficulties in understanding the disease and the guidelines for its treatment and prevention^20^.

Considering this panorama, the health professional must be able to clarify these situations in complementary ways, seeking ways to increase the effectiveness of TB control and promote a holistic view of health care^21^.

We observed the predominance of the pulmonary clinical form of the disease, which was expected, since it is its most common form^1,2^. Most cases reached a cure (75.3%), however, this rate was far below the WHO recommendation of 85%^1^. Our findings point to the high number of dropouts (12.5%), above that established by the WHO (5%), which is a predictor for the development of resistant tuberculosis, whose treatment is longer and more costly for the health system^20^.

Similarly, we observed a high percentage of uninformed variables, such as the comorbidities HIV, diabetes, alcoholism and mental illnesses; which is information of the utmost relevance for the clinical management of cases. Some assumptions for non-records are the non-prioritization of this information in the clinic, the incipient qualification of the teams on the importance of health surveillance based on the quality of the data, and the lack of evaluation and feedback to the teams about the data produced in the units in which the diagnoses were made.

In the studied period, the incidence of tuberculosis was decreasing, a pattern that follows the worldwide trend^1,22^. However, this decline occurs at a speed below the WHO expectations^1^, thus it is unlikely that the disease will be fully erradicated by 2050.

With ITS, we identified no change of level after the implementation of RMT-TB, however, the period of “post-intervention” was classified as increasing, confirming the hypothesis that the test, thanks to its high sensitivity^23–25^, may have influenced the increase of cases in the municipality.

Bacilloscopy remains in the Unified Health System (SUS) as a method for monthly follow-up of treatment and RMT-TB is performed only for initial diagnosis. The culture, considered the gold standard for the diagnosis, takes around 45 to 60 days, due to slow replication of the bacillus, which impedes its use as the only strategy for early diagnosis and timely therapy^4,5^.

However, the use of RMT-TB does not dispense with the culture, which should always be considered, so that its data are compared with those of RMT-TB (subject to false positives regarding the sensitivity or rifampicin resistance) and drug-resistant strains are identified^24^.

The main strategy for tuberculosis control is the early detection of cases – especially bacilliferous pulmonary cases, which have greater epidemiological importance – aiming at maintaining the transmission chain of the disease, followed by adequate treatment and complete cure^26^. According to the WHO^27^, about 43 million deaths were avoided between the years 2000 and 2014 by the use of early diagnosis and appropriate treatment. Thus, strategies and public policies with this purpose can, in the medium or long term, reduce disease rates. This means reliable means of diagnosis are essential.

Our study also evidenced the seasonality of TB, with significant oscillations of cases and incidence throughout the year, thus confirming one of our initial hypotheses. The findings showed that the incidence increased between December and June, coinciding with the rainy season. Respiratory diseases in the Eastern Amazon^7^ showed this behavior, but it had not yet been evidenced for TB.

This can be attributed to the need for people to stay on overcrowded places with little ventilation in these periods. In warmer times, on the other hand, the temporal trend decreased, since people seek more airy places, which may decrease the contagion with the bacillus causing TB.

The use of Kernel and scan statistics made possible to identify that the highest densities of cases and risk areas (SC4) are in the Central, Northern and Southern districts and in the regions that concentrate the lowest Human Development Indexes of the municipality, which, added to the existence of bridge areas (stilt houses), to the absence of sanitation, to the clusters of population, and to the large number of informal workers corroborate the spread of the disease.

Near those locations, other areas considered as protection (SC1, SC2 and SC3) were identified, which leads to the assumption that these regions have undiagnosed cases. Therefore, underreporting is a possibility, since social vulnerability is also notable in these regions. This result should be cautiously analyzed and serves as a warning to health surveillance teams.

The use of strategies such as income transfer and/or compensatory policies, to alleviate social inequality and its deleterious effects on the vulnerable population is critical^28^.

Among the limitations of this study, the ecological fallacy stands out^29^, meaning the data are analyzed at the aggregate level and the results cannot be interpreted at the individual level. Also note the use of secondary data, which may contain typing errors and missing information, which may interfere with the analyses^30^. Another limitation was the lack of the socioeconomic variable income, which could be used as a covariate in the adjustment of the temporal model.

The use of two distinct approaches – time series and spatial analyses – to understand the behavior of tuberculosis in the eastern Amazon makes this study relevant. The evidence of the influence of RMT-TB on TB incidence rates and of its seasonal behavior in the Eastern Amazon adds to its originality, making this a possible reference for future studies.

This will also help orient public policies, given that differences in of TB load between the territories are evidenced, with social vulnerability as one of its probable explanations.
