# Supplementary material for: Temporal trend of tuberculosis incidence and its spatial distribution in Macapá – Amapá
Source: Rev Saude Publica. 2021 Nov 18;55:96. doi: 10.11606/s1518-8787.2021055003431 (PMC8647990; doi:10.11606/s1518-8787.2021055003431)
Supplement: Translation

# Translation: Tendência temporal da incidência de tuberculose e sua distribuição espacial em Macapá-AP

**Authors:** Clóvis Luciano Giacomet, Marcio Souza Santos, Thaís Zamboni Berra, Yan Mathias Alves, Luana Seles Alves, Fernanda Bruzadelli Paulino da Costa, Antonio Carlos Vieira Ramos, Juliane de Almeida Crispim, Aline Aparecida Monroe, Ione Carvalho Pinto, Regina Célia Fiorati, Marcos Augusto Moraes Arcoverde, Dulce Gomes, Giselle Lima de Freitas, Mellina Yamamura, Ricardo Alexandre Arcêncio

**Affiliations:** I Ribeirão Preto SP Brasil Universidade de São Paulo. Escola de Enfermagem de Ribeirão Preto. Ribeirão Preto, SP, Brasil; II Foz do Iguaçu PR Brasil Universidade do Oeste do Paraná, Escola de Enfermagem. Foz do Iguaçu, PR, Brasil; III Évora Portugal Universidade de Évora. Departamento de Matemática. Évora, Portugal; IV Belo Horizonte MG Brasil Universidades Federal de Minas Gerais. Faculdades de Enfermagem. Belo Horizonte, MG, Brasil; V São Carlos SP Brasil Universidades Federal de São Carlos. Faculdades de Enfermagem. São Carlos, SP, Brasil

**Keywords:** Tuberculose, epidemiologia, Conglomerados Espaço-Temporais, Análise Espacial, Estudos Ecológicos

## Abstract

**OBJETIVO:**

Avaliar a tendência temporal da incidência da tuberculose após a implementação do teste rápido molecular, identificar se a tuberculose apresenta variação sazonal e classificar o território de acordo com a densidade de casos e as áreas de risco em Macapá-AP.

**MÉTODOS:**

Estudo ecológico composto por casos de tuberculose registrados no SINAN entre 2001 e 2017. Foi utilizado o teste Prais-Winsten para classificar a tendência temporal da incidência e a Série Temporal Interrompida para identificar mudanças na tendência temporal antes e depois da implementação do teste rápido molecular, além de verificar a sazonalidade no município. Utilizou-se o estimador de Kernel para classificar a densidade de casos e estatística de varredura para identificar áreas de risco da tuberculose.

**RESULTADOS:**

Foram identificados 1730 casos, observando-se que a tendência temporal da incidência de tuberculose foi decrescente (-0,27% por mês, IC95% −0,13 a −0,41). Não houve mudança de nível na série temporal após a implantação do teste molecular GeneXpert^®^ MTB/RIF, porém, o período pós teste foi classificado como crescente em termos da incidência (+2,09% por mês, IC95% 0,92 a 3,27). Quanto à variação sazonal, apresentou crescimento (+13,7%/mês, IC95% 4,71 a 23,87) nos meses de dezembro a junho, referente ao período de chuvas – chamado inverno amazônico – e decréscimo (-9,21% por mês, IC95% −1,37 a −16,63) nos demais períodos. Por meio de Kernel, foram classificadas áreas com alta densidade de casos nos distritos Central e Norte e, com a estatística de varredura, foram identificados três aglomerados de proteção, AE1 (RR = 0,07), AE2 (RR = 0,23) e AE3 (RR = 0,36), e um aglomerado de alto risco, AE4 (RR = 1,47).

**CONCLUSÃO:**

A tendência temporal da incidência de tuberculose se revelou decrescente na série temporal, todavia, um crescimento na detecção foi observado após introdução do TRM-TB, e ainda se evidenciou que há comportamento sazonal da tuberculose. A distribuição dos casos foi heterogênea, com tendência de concentração em territórios vulneráveis e de risco, evidenciando um padrão de desigualdade da doença no território.

## INTRODUÇÃO

A tuberculose permanece como um problema de saúde pública mundial. Segundo a Organização Mundial da Saúde (OMS), é uma das 10 principais causas de morte no mundo e a principal causa de morte por um único agente infeccioso em pessoas vivendo com o vírus da imunodeficiência humana (PVHIV). Em 2019, cerca de 10 milhões de pessoas adoeceram por tuberculose no mundo e cerca de 1,4 milhões de pessoas morreram em decorrência da doença^1^.

No Brasil, em 2019, foram diagnosticados 73.864 casos novos de tuberculose (incidência de 35,0 casos/100 mil habitantes) e vale destacar que a região Norte dos pais é a mais acometida pela doença^2^. No Amapá, em 2019, foram diagnosticados 296 casos novos da doença distribuídos em 14 dos 16 municípios que compõem o estado, sendo Macapá a cidade com maior número de casos, que registrou um aumento de 23,9% em relação a 2018^3^.

Para sua eliminação, são necessárias ações de maior amplitude, como a busca ativa de sintomáticos respiratórios visando ao diagnóstico precoce de casos e ao início rápido do tratamento, de modo a evitar que a pessoa com tuberculose permaneça na fase bacilífera e transmita a doença para outras pessoas e comunidades^4^.

No que tange ao diagnóstico, o teste rápido molecular para tuberculose (TRM-TB), realizado através do sistema GeneXpert^®^ MTB/RIF, consiste em um exame automatizado, que reduz o tempo de resultado do diagnóstico para no máximo 2 horas e gera economia nos custos, incluindo aqueles decorrentes de internações resultantes da evolução da doença e do diagnóstico tardio. Os custos de internação devido à tuberculose elevam em até cem vezes com relação àqueles de tratamento ocorrido em nível ambulatorial^5^.

Com resultados mais rápidos e apresentando alta sensibilidade e especificidade, respectivamente de 98% e 88%^5^, o tratamento pode ser iniciado precocemente, sem a necessidade de aguardar a confirmação pela cultura (que pode demorar até 60 dias).

A aplicação do TRM-TB ocorreu após a validação de seus resultados pela OMS, em 2010, quando a tecnologia diagnóstica passou a ser recomendada e adotada em vários sistemas de saúde em todo o mundo. No Brasil, a Comissão Nacional de Incorporação de Tecnologias do SUS (CONITEC-SUS) e a Agência Nacional de Vigilância Sanitária (ANVISA) aprovaram a utilização do teste no ano de 2013, quando foram adquiridos 160 equipamentos do laboratório Cepheid pelo Ministério da Saúde, distribuídos estrategicamente pelo país^5^.

Levanta-se a hipótese de que o TRM-TB, por sua alta sensibilidade, modificou o número de casos diagnosticados e contribuiu com a elucidação de casos, especificamente dos paucibacilares, antes não detectados pela baciloscopia. Ou seja, investiga-se se o TRM-TB pode ter mudado o panorama epidemiológico da tuberculose.

Além da elevação de casos, tem-se como outra hipótese do estudo a variação sazonal da doença, fenômeno que tem sido observado em estudos realizados em vários países^6^, todavia pouco explorado no Brasil. Outro estudo^7^ evidenciou a ocorrência de elevação das doenças respiratórias na Amazônia nos períodos de inverno, quando aumentam as chuvas na região e, por isso, levanta-se a hipótese de que esse aumento nos índices possa acontecer também com a tuberculose.

Diante do exposto, o estudo buscou avaliar a tendência temporal da incidência da tuberculose após a implementação do TRM-TB, identificar se ela apresenta variação sazonal e classificar o território de acordo com densidade de casos e áreas de risco para a doença em município da Amazônia Oriental.

## MÉTODOS

### Delineamento do Estudo

Estudo ecológico^8^ realizado em Macapá, capital do estado do Amapá, que está inserido na Amazônia Oriental e faz divisa com a Guiana Francesa.

### Local de Estudo

A cidade de Macapá conta com uma área de aproximadamente 6.563,849km², densidade demográfica de 62,14hab/km² e população estimada de 512.902 pessoas em 2020. Está administrativamente dividida em quatro distritos: Norte, Oeste, Centro e Sul^9^.

O diagnóstico dos casos de tuberculose em Macapá segue o protocolo do Ministério da Saúde^5^. Dessa forma, todas as Unidades Básicas de Saúde (UBS) recebem casos suspeitos que, posteriormente, são encaminhados para a unidade de referência do estado, chamada Centro Regional de Doenças Tropicais (CRDT). Trata-se de um serviço centralizado para todo o estado, que realiza acompanhamento dos casos e dispensação dos medicamentos.

O estado conta ainda com o Laboratório Central (LACEN), que realiza os exames laboratoriais, incluindo aqueles para o diagnóstico de tuberculose. No LACEN, encontra-se o único equipamento GeneXpert^®^ MTB/RIF, referência para o diagnóstico de tuberculose, no Amapá. Ressalta-se ainda que a implementação do TRM-TB ocorreu em setembro de 2014, que foi considerado o ano de corte nas séries temporais^5^.

### População

A população do estudo foi composta por casos de tuberculose notificados no Sistema de Notificação de Agravos de Notificação (SINAN) entre 2001 e 2017.

### Plano de Análise

Inicialmente, com o objetivo de caracterizar o perfil dos casos de tuberculose notificados no município em estudo, foram calculadas as frequências absolutas e relativas das variáveis apresentadas na ficha de notificação através do *software* IBM SPSS Statistics versão 25.

Para classificar a tendência temporal da incidência de tuberculose, a taxa foi calculada de modo mensal, considerando o número absoluto de casos no numerador e a população no denominador, com fator de multiplicação por 1000 habitantes. As taxas de incidência foram logaritmizadas (log10) de modo a estabilizar a variância ao longo do tempo^10^. Os gráficos da distribuição dos casos e das taxas estimadas ao longo da série temporal foram produzidos por meio do *software* RStudio.

Utilizou-se o método de autorregressão Prais-Winsten através do *software* STATA para classificar a tendência temporal da incidência de tuberculose crescente, decrescente ou estacionária e, se crescente ou decrescente, foi calculada a porcentagem de variação mensal (MPC – *monthly percent change*) e seu respectivo intervalo de confiança de 95% (IC95%)^10^.

Com o objetivo de verificar se houve mudança na tendência temporal da incidência de tuberculose após a implementação do TRM-TB e se a doença apresenta variação sazonal, recorreu-se à Série Temporal Interrompida (STI), definida como o mais efetivo recurso para avaliar o impacto de uma intervenção, possibilitando verificar se há influência imediata (mudança de nível) e/ou influência progressiva (mudança de tendência) nos valores da série^11^.

Para aplicação da STI, foram consideradas as taxas de incidência mensais logaritmizadas (Log10) anteriormente calculadas. A mudança de nível foi chamada de “intervenção” e a mudança progressiva, de “pós-intervenção”^11^. Além disso, foram incorporadas nas análises as funções trigonométricas seno e cosseno, de modo a verificar se a tuberculose apresenta variação sazonal^10^. Para a STI, também foi utilizado o *software* STATA versão 14.

Na primeira etapa, como critérios de inclusão, foram considerados todos os casos notificados de tuberculose de residentes na área urbana do município de Macapá. Para os casos notificados mais de uma vez no sistema, considerou-se a notificação mais atual. É importante destacar que, no período analisado, todos os casos arrolados ao estudo são de novas notificações no SINAN e, portanto, foi a incidência estimada.

Para classificar a densidade de casos, recorreu-se ao estimador de densidade de Kernel. Inicialmente, foi necessário realizar a geocodificação dos casos mediante obtenção das coordenadas geográficas (latitude e longitude) dos endereços residenciais dos casos notificados, utilizando a ferramenta Google Earth Pro.

O georreferenciamento dos casos incluídos no estudo foi realizado através do *software* ArcGis 10.5, considerando os setores censitários urbanos do município de Macapá como unidade de análise e a população residente por setor censitário, como configuração para padronização da análise.

Destaca-se que, na fase da análise espacial, foram excluídos os casos de pessoas em situação de rua, indivíduos privados de liberdade, indígenas ou pessoas cujo endereço de notificação era de órgãos municipais (hospitais ou unidades de saúde) devido à impossibilidade de obtenção das coordenadas geográficas dos endereços residenciais.

Utilizou-se o estimador de intensidade de Kernel, a partir do *software* ArcGis 10.5, que consiste em um método de interpolação exploratória que define áreas circulares de influência em torno de pontos de ocorrências de um fenômeno, gerando uma densidade superficial para identificação de áreas vulneráveis^12^. Assim, considerando um raio de 1.000 metros^13,14^, os mapas temáticos da distribuição da densidade dos casos de tuberculose também foram gerados no *software* ArcGIS 10.5.

Por fim, para identificar as áreas de risco para tuberculose em Macapá, foi utilizada a estatística de varredura^15^, em que a procura por aglomerados espaciais é realizada através de um círculo de raio variável em torno do centroide de cada unidade de análise, que, no presente estudo, foram os setores censitários. Na técnica, é calculado o número de casos observados e esperados dentro de cada círculo, procedimento realizado até que todos os centroides sejam testados. Quando o valor observado na área delimitada pelo círculo for maior ou menor do que o esperado, o círculo é chamado de ou aglomerado^16^.

Nessas análises, adotou-se as seguintes características: modelo discreto de Poisson; não existir sobreposição geográfica dos aglomerados; aglomerados com formato circular; 999 replicações na simulação de Monte Carlo; e tamanho da população exposta estipulado pelo coeficiente de Gini, em que o número de casos é comparado aos dados da população de base e o número esperado de casos em cada setor censitário é proporcional ao tamanho da população em risco^16,17^. Além disso, o risco relativo (RR) e intervalo de confiança (IC95%) de cada aglomerado foram calculados. Destaca-se que foram considerados aglomerados estatisticamente significativos aqueles com p < 0,05.

O estudo foi aprovado pelo Comitê de Ética em Pesquisa da Escola de Enfermagem de Ribeirão Preto, com Certificado de Apresentação para Apreciação Ética (CAAE) n° 23043019.2.0000.5393.

## RESULTADOS

Entre 2001 e 2017, foram notificados 1.730 casos de tuberculose em Macapá, com idade mínima de um ano e máxima de 89 anos.

Pode-se observar que a maioria dos casos foi do sexo feminino (59,3%), com idade entre 31 e 59 anos (47,6%), raça/cor parda (67,9%) e com ensino fundamental incompleto (46,6%). Observa-se também a predominância da forma pulmonar (85,1%).

Conforme a Tabela 1, observa-se o alto número de informações em branco e/ou ignoradas referente à escolaridade (16,4%), coinfecção TB-HIV (50%), TB-Diabetes (41,8%), alcoolismo (39,7%) e doença mental (41,7%). Não obstante, essas informações são relevantes para o manejo clínico dos casos e das condutas.

**Tabela 1:** Perfil sociodemográfico e clínico-epidemiológico dos casos de tuberculose (TB), notificados em Macapá-AP, Brasil (2001 a 2017).

Variáveis		n (1.730) (%)	
Idade (anos)		
	0 a 14 anos	74 (4,3)
	15 a 30 anos	640 (37,0)
	31 a 59 anos	823 (47,6)
	≥ 60 anos	175 (10,1)
	Ignorado/branco	18 (1,0)
Sexo		
	Masculino	704 (40,7)
	Feminino	1.026 (59,3)
Raça		
	Branco	325 (18,3)
	Preto	124 (7,7)
	Amarela	28 (1,6)
	Parda	1.175 (67,9)
	Indígena	14 (0,8)
	Ignorado/branco	64 (3,7)
Escolaridade		
	Analfabeto	128 (7,4)
	Ensino fundamental completo	112 (6,5)
	Ensino fundamental incompleto	803 (46,6)
	Ensino médio completo	224 (12,9)
	Ensino superior incompleto	52 (3,0)
	Educação superior completo	127 (7,3)
	Ignorado/branco	284 (16,4)
Forma		
	Extrapulmonar	236 (13,6)
	Pulmonar	1.472 (85,1)
	Pulmonar + Extrapulmonar	18 (1,0)
	Em branco/Ignorado	4 (0,3)
Desfecho		
	Cura	1.303 (75,3)
	Abandono	217 (12,5)
	Óbito por tuberculose	17 (1,0)
	Óbito por outra causa	47 (2,7)
	Transferência/mudança de país	108 (6,3)
	Mudança de diagnóstico	30 (1,7)
	TB multirresistente	04 (0,2)
	Mudança de esquema por intolerância medicamentosa	03 (0,2)
	Mudança de diagnóstico	01 (0,1)
Coinfecção TB-HIV		
	Não	787 (45,5)
	Sim	77 (4,5)
	Ignorado/Em branco	866 (50,0)
Coinfecção TB-diabetes		
	Não	912 (52,8)
	Sim	94 (5,4)
	Ignorado/Em branco	724 (41,8)
Alcoolismo		
	Não	896 (51,7)
	Sim	148 (8,6)
	Ignorado/Em branco	686 (39,7)
Doença mental		
	Não	1.001 (57,8)
	Sim	12 (0,7)
	Ignorado/Em branco	717 (41,5)

A Figura 1 apresenta a série temporal do número de casos e da incidência de tuberculose ao longo dos meses de 2001 a 2017, em que é possível observar a presença de picos e quedas por todo o período em estudo. Conclui-se, portanto, que a série inicia-se com números mais elevados, sofre uma queda e finaliza de maneira crescente.

**Figura 1:** Série temporal do número de casos e da incidência de tuberculose em Macapá-AP, Brasil (2001 a 2017).

A Tabela 2 apresenta os resultados das técnicas de Prais-Winsten e STI. Foi possível classificar a tendência temporal da incidência de tuberculose em Macapá como decrescente, com queda de -−.27%/mês (IC95% -−.41 a -−.13).

**Tabela 2:** Tendência temporal, impacto do diagnóstico por meio do teste rápido molecular e variação sazonal na incidência de casos de tuberculose, Macapá – AP, Brasil (2001 a 2017).

Prais-Winsten			
	Coeficiente (IC95%)	Tendência	MPC (IC95%)
Tuberculose em Macapá	-0,001 (-0,006 a -−,000)	Decrescente	-0,27 (-0,41 a -−,13)
**Série temporal interrompida (STI)**			
	Coeficiente (IC95%)	Tendência	MPC (IC95%)
Intervenção	1,014 (3,001 a -−,971)	Estacionário	NA
Pós-Intervenção	0,009 (0,014 a 0,004)	Crescente	2,09 (3,27 a 0,92)
Seno	0,056 (0,093 a 0,020)	Crescente	13,76 (23,87 a 4,71)
Cosseno	-0,042 (-0,079 a -−,006)	Decrescente	-9,21 (-16,63 a -1,37)

MPC: *monthly percent change*;

NA: não se aplica.

Também foi identificado que não houve mudança de nível (intervenção) na série temporal após a implementação do TRM-TB. Todavia, o período pós implantação do TRM-TB foi classificado como crescente, com aumento de 2,09%/mês (IC95% 3,27 a 0,92), indicando que houve um aumento progressivo na incidência da tuberculose após a implementação do teste.

Quanto à sazonalidade, foi possível identificar que a doença em Macapá apresenta crescimento de 13,7%/mês (IC95% 23,87 a 4,71) nos meses entre dezembro e junho (seno), período coincidente com as chuvas ocorridas no chamado inverno amazônico. Outrossim, identificou-se decréscimo de 9,21%/mês (IC95% −1,37 a Ȓ16,63) nos meses restantes (cosseno), que são épocas mais quentes e de estiagem.

Dos 1730 casos de tuberculose identificados, 59 (3,41%) foram excluídos por não possuírem endereço. Dos 1.671 casos restantes, 1475 (88,2%) tiveram as coordenadas geográficas identificadas e, portanto, georreferenciadas.

Com o estimador de Kernel, pode-se observar áreas classificadas como muito alta densidade de casos nos distritos Central (bairros Laguinho e Perpétuo Socorro) e Norte (bairros Pacoval e Cidade Nova), com variação de 70,67 a 88,33 casos/km². Áreas classificadas como alta densidade de casos estão nos distritos Sul (bairro Buritizal), Centro (bairros Laguinho e Perpétuo Socorro) e Norte (bairros Pacoval e Cidade Nova), com variação de 53,01 a 70,66 casos/km^2^, conforme Figura 2.

**Figura 2:** Localização geografia dos casos de tuberculose notificados em Macapá-AP, Brasil (2001 a 2017).

A estatística de varredura possibilitou a identificação de quatro aglomerados espaciais (AE) de risco para a tuberculose em Macapá (Figura 3), considerando o parâmetro de 40% da população exposta, segundo o índice de Gini.

**Figura 3:** Áreas de risco espacial para tuberculose em Macapá-AP, Brasil (2001 a 2017).

O AE1, considerado de proteção (RR = 0,07; IC95% 0,01 a 0,48), foi formado por seis setores censitários (p = 0,03) do distrito Sul, com população de 3.799 habitantes, um caso observado e 14 casos esperados.

O AE2, também de proteção (RR = 0,23; IC95% 0,10 a 0,52), foi composto por 10 setores censitários (p = 0,03) do distrito Sul, com população de 6.578 habitantes, seis casos observados e 25 casos esperados.

O AE3, também considerado de proteção (RR = 0,36; IC95% 0,20 a 0,59), foi formado por 11 setores censitários do distrito Oeste (p = 0,04), com 10.172 habitantes, 14 casos observados e 38 casos esperados.

Por fim, o AE4 (p < 0,01), considerado de risco para a tuberculose (RR = 1,47; IC95% 1,39 a 1,72), foi formado por 146 setores censitários dos distritos Norte, Sul e Centro, com população de 122.356 habitantes, 620 casos observados e 487 casos esperados.

## DISCUSSÃO

Foi possível traçar o perfil sociodemográfico e clínico-epidemiológico dos casos de tuberculose em Macapá-AP. Com o cálculo da incidência mensal numa série temporal de 16 anos, foi possível classificar a tendência temporal e identificar mudanças na série após a implementação do TRM-TB, além de evidenciar que a tuberculose apresenta variação sazonal. Por fim, foi possível classificar áreas de acordo com a densidade de casos e identificar áreas de risco e proteção em Macapá-AP.

A maioria das pessoas acometidas por tuberculose em Macapá era do sexo feminino, resultado diferente dos achados da literatura, haja vista a evidência de que a maioria dos casos geralmente ocorre em pessoas do sexo masculino, evidência explicada pelo fato de que o homem tende a minimizar seu autocuidado e a estar mais exposto aos fatores de risco para a doença^18^.

A predominância do sexo feminino pode estar relacionada ao fato de as mulheres serem chefes de família, subvalorizadas no mercado de trabalho e de desempenharem, grande parte das vezes, tripla jornada de trabalho. O que são fatores estressantes que podem ser considerados de risco para o adoecimento^18^. Além disso, destaca-se a desigualdade de gênero, herdada de questões culturais locais dos povos ribeirinhos, em que a mulher é vista como coadjuvante e o homem, como provedor, características ainda presentes no cenário de estudo.

A maioria dos casos foi detectada em pessoas adultas em faixa etária economicamente ativa, fase da vida em que os indivíduos tendem a enfrentar maiores aglomerações, por conta do trabalho e das atividades cotidianas, aumentando as chances de adoecimento^19^.

Pessoas pardas apresentaram mais propensão à doença no estudo, o que pode ser justificado por ser a raça/cor predominante no município. Quase 52% da população Macapá se autodeclara parda e, neste estudo, os autodeclarados pardos são 67,9%, o que justificaria os altos índices da tuberculose nessa população.

A maioria dos casos notificados possui ensino fundamental incompleto, que é um indicador indireto de condições sociais acarretadas pelo contexto social no qual a população está inserida. Esse dado representa um entrave para o controle da tuberculose, visto que esses indivíduos poderão ter dificuldades na compreensão da doença e das orientações para seu tratamento e prevenção^20^.

Mediante esse panorama, o profissional de saúde deve estar apto a esclarecer essas situações de formas complementares, buscando maneiras de aumentar a efetividade do controle da tuberculose, bem como uma visão holística do cuidado em saúde^21^.

Também foi observada a predominância da forma clínica pulmonar, o que era esperado, visto que é a forma mais comum da doença^1,2^. A maioria dos casos teve cura (75,3%), todavia, essa taxa ficou muito abaixo da recomendação da OMS, de 85%^1^. Os achados acenam para o elevado número de abandonos (12,5%), acima do estabelecido pela OMS (5%), que é fator preditor para desenvolvimento da tuberculose resistente, cujo tratamento é mais longo e mais oneroso para o sistema de saúde^20^.

Similarmente, foi evidenciado um alto percentual de variáveis em branco, como as comorbidades de HIV, diabetes, alcoolismo e doenças mentais; essas informações são de suma relevância para o manejo clínico dos casos. Alguns pressupostos para os não-registros são a não priorização dessas informações na clínica, a qualificação incipiente das equipes sobre importância da vigilância em saúde baseada na qualidade dos dados, e a falta de avaliação e devolutiva às equipes sobre os dados produzidos nas unidades nas quais os diagnósticos foram realizados.

No período estudado, a incidência da tuberculose foi decrescente, padrão que segue a tendência mundial^1,22^. Porém, esse declínio ocorre em velocidade aquém das expectativas da OMS^1^ e, desse modo, é pouco provável que se alcance a eliminação da doença até 2050.

Com a STI, não foi identificada mudança de nível após a implementação do TRM-TB, porém, o período de “pós-intervenção” foi classificado como crescente, confirmando a hipótese de que o teste, por sua alta sensibilidade^23–25^, possa ter influenciado no aumento de casos no município.

A baciloscopia permanece no SUS como método para acompanhamento mensal do tratamento, sendo o TRM-TB realizado somente para o diagnóstico inicial. A cultura, considerada padrão-ouro, leva em torno de 45 a 60 dias, devido à replicação lenta do bacilo, o que impossibilita ser utilizada como única estratégia para diagnóstico precoce e terapêutica oportuna^4,5^.

Todavia, o uso do TRM-TB não prescinde o da cultura, que deve ser considerada de forma universal, para que seus dados sejam confrontados com os do TRM-TB (passível de falsos positivos quanto à sensibilidade ou resistência da rifampicina) e, além disso, triar cepas resistentes aos medicamentos^24^.

A principal estratégia para o controle da tuberculose é a detecção precoce dos casos, principalmente os casos pulmonares bacilíferos, que possuem maior importância epidemiológica, visando à manutenção da cadeia de transmissão da doença, seguida pelo tratamento adequado e alcance da cura^26^. Segundo a OMS^27^, entre os anos de 2000 e 2014, foi possível evitar o óbito de cerca de 43 milhões de pessoas através de diagnóstico precoce e tratamento adequado. Dessa maneira, entende-se que estratégias e políticas públicas que tenham essa finalidade podem, a médio ou longo prazo, propiciar a diminuição dos índices da doença e, frente a isso, meios confiáveis de diagnóstico são essenciais.

Outra evidência trazida é a sazonalidade da tuberculose, verificando-se oscilações significativas de caso e incidência ao longo do ano, confirmando, portanto, uma das hipóteses do estudo. Os achados evidenciaram que a incidência aumenta entre dezembro e junho, coincidentemente com os períodos de chuvas. Esse comportamento foi observado nas doenças respiratórias na Amazônia Oriental^7^, mas ainda não tinha sido evidenciado no contexto da tuberculose.

Esse fato pode ser atribuído à necessidade de as pessoas concentrarem-se em ambientes superlotados e com pouca ventilação nos períodos em questão. Em contraste a isso, em épocas mais quentes, a tendência temporal apresentou decréscimo, visto que as pessoas procuram ficar em ambientes mais arejados, o que pode diminuir o contágio do bacilo causador da tuberculose.

Com o uso do Kernel e estatística de varredura, foi possível identificar que as maiores densidades de casos e áreas de risco (AE4) estão nos distritos Central, Norte e Sul e nas regiões que concentram os menores Índices de Desenvolvimento Humano do município, que, somados à existência de áreas de ponte (casas de palafita), à ausência de saneamento, aos aglomerados populacionais e ao grande número de trabalhadores informais, em conjunto, corroboram para a disseminação da doença.

Próximo às localidades acima referidas, foram identificadas outras áreas consideradas de proteção (AE1, AE2 e AE3), o que leva à suposição de que há casos não diagnosticados nessas regiões. Portanto, há a possibilidade de subnotificação, visto que a vulnerabilidade social é também marcante nessas regiões. Esse resultado deve ser analisado com a devida cautela e serve de alerta para as equipes de vigilância em saúde.

Considera-se importante a adoção de estratégias como políticas de transferência de renda e/ou compensatórias, visando aliviar a desigualdade social e seus efeitos deletérios na população vulnerável^28^.

Dentre as limitações deste estudo, destaca-se a falácia ecológica^29^, em que os dados são analisados em nível agregado e seus resultados não podem ser interpretados em nível individual. Cabe também mencionar o uso de dados secundários, que podem conter falhas de digitação e informações em branco, que podem interferir nas análises^30^. Outra limitação foi a não existência da variável socioeconômica renda, que poderia ser utilizada como covariável no ajustamento do modelo temporal.

O estudo avança no conhecimento na medida em que se utiliza de duas abordagens distintas para a compreensão do comportamento da tuberculose na Amazônia Oriental, das séries temporais e de análises espaciais. Acresce à sua originalidade a evidência da influência do TRM-TB nas taxas de incidência da tuberculose. Seu ineditismo reside, também, no fato de evidenciar seu comportamento sazonal na Amazônia Oriental, antes não conhecido naquele cenário.

A investigação pode se tornar uma referência para investigações futuras intencionadas na compreensão de agravo. Ajudará, também, na orientação de políticas públicas, haja vista que ficam evidenciadas as diferenças nos territórios em termos da carga da tuberculose, sendo a vulnerabilidade social uma das prováveis explicações para esse cenário.
